# Mindfulness Meditation Activates Altruism

**DOI:** 10.1038/s41598-020-62652-1

**Published:** 2020-04-16

**Authors:** Sage K. Iwamoto, Marcus Alexander, Mark Torres, Michael R. Irwin, Nicholas A. Christakis, Akihiro Nishi

**Affiliations:** 1 Present Address: College of Letters & Sciences, UC Berkeley, Berkeley, CA 94720 United States; 2 Los Angeles Center for Enriched Studies, Los Angeles, CA 90035 United States; 3 Human Nature Lab, Yale Institute for Network Science, Yale University, New Haven, CT 06514 United States; 4 Mindful Awareness Research Center and Cousins Center for Psychoneuroimmunology, Jane and Terry Semel Institute for Neuroscience at UCLA, and Department of Psychiatry and Biobehavioral Sciences, David Geffen School of Medicine at UCLA, Los Angeles, CA 90095 United States; 5 Departments of Sociology, Ecology and Evolutionary Biology, Medicine, Statistics and Data Science, and Biomedical Engineering, Yale Institute for Network Science, P.O. Box 208263, New Haven, CT 06520-8263 United States; 6 Department of Epidemiology, UCLA Fielding School of Public Health, Los Angeles, CA 90095 United States

**Keywords:** Cultural evolution, Human behaviour

## Abstract

Clinical evidence suggests that mindfulness meditation reduces anxiety, depression, and stress, and improves emotion regulation due to modulation of activity in neural substrates linked to the regulation of emotions and social preferences. However, less was known about whether mindfulness meditation might alter pro-social behavior. Here we examined whether mindfulness meditation activates human altruism, a component of social cooperation. Using a simple donation game, which is a real-world version of the Dictator’s Game, we randomly assigned 326 subjects to a mindfulness meditation online session or control and measured their willingness to donate a portion of their payment for participation as a charitable donation. Subjects who underwent the meditation treatment donated at a 2.61 times higher rate than the control (p = 0.005), after controlling for socio-demographics. We also found a larger treatment effect of meditation among those who did not go to college (p < 0.001) and those who were under 25 years of age (p < 0.001), with both subject groups contributing virtually nothing in the control condition. Our results imply high context modularity of human altruism and the development of intervention approaches including mindfulness meditation to increase social cooperation, especially among subjects with low baseline willingness to contribute.

## Introduction

Cooperation is an essential behavior in constructing and maintaining human societies and the past two decades of cognitive neuroscience and behavioral economics experiments have improved our understanding of neurophysiological bases of such behavior. Neuroimaging suggests that cooperation is associated with reward-processing brain areas including rostral anterior cingulate cortex (rACC)^1^, which is known to modulate fear processing in amygdala^2^; as well as the ventromedial prefrontal cortex (vmPFC), nucleus accumbens (NACC), and caudate^3–5^. Recent research gives us an even more granular view of the pathways involved, showing that “pay-it-forward” indirect reciprocity (altruism without expectation of returned favor, which underlies large-scale cooperation, specifically based on empathy and other-regarding social preferences rather than reputation) is associated with activation of the anterior insula (AI), which in turn regulates the caudate^6^.

Cooperation can be generally defined as individual behavior whereby “one individual pays a cost for others to receive a benefit”^7^. Cooperation can be further modelled as equilibrium of games where subjects still act in their self-interest but their utility function (a function that defines individual preferences of given scenarios) includes social preferences that have been modelled in various ways, such as inequity aversion, a second utility function over others’ well-being, etc^8–10^. In the present study we focus on the act of making a private decision regarding what proportion of payment to keep for oneself and what proportion to make as a charitable donation, which is a form of indirect reciprocity or generalized altruism.

Evidence also suggests that certain types of social environments, social cues and stimuli can alter the people’s perception on social dilemma (i.e. individual cost vs. collective benefit) and can enhance human cooperation. For example, people are more cooperative when they connect with cooperative individuals than when they connect with non-cooperative individuals^11^. Providing reputation information of others (how cooperative they are in the past) can contribute to constructing a cooperative social norm in social networks^12,13^. It is also known that a time pressure (study participants are asked to decide cooperate or defect within certain seconds in economic games) can improve the level of cooperation^14^. Another study found that the introduction of integrative institutions activates altruism and improves cooperation among deeply divided ethnoreligious groups^15^. A series of studies found that an exposure to oxytocin through nasal intake improved the level of cooperation^16,17^.

Meditation is “a form of mental training that aims to improve an individual’s core psychological capacities, such as attentional and emotional self-regulation”^18^, which has gained popularity as a focus of research over the past decade^19^. Meditation has a wide range of types such as loving kindness meditation (taught by spiritual leaders or experts versed in the Buddhist tradition, focused on silent repetition of phrases based on Buddhist teachings, and incorporated into holistic health and group support programs) and mindfulness meditation (increasingly taught by trained clinical psychologists, focused on emotional self-regulation and focusing attention, and incorporated into cognitive therapy and clinical care). Meditation both over short-term and long-term has been found to improve cognition^20–22^. One study of 54 college students in Singapore playing the Dictator Game (where the Dictator, player A, divides money provided by the experimenter between what she wants to keep for herself and what she wants to give away to an anonymous player B) reported that loving kindness meditation led to higher cooperation^23^.

Mindfulness meditation emphasizes non-judgmental attention to experiences in the present moment^24^. Meditation practices mainly modulate brain activities responsible for cognitive control, emotion regulation, and empathy, each of which is associated with specific brain areas including the anterior cingulate cortex (ACC), insula, and amygdala^25–28^.

A recent meta-analysis of clinical data concludes that mindfulness meditation practices have a positive effect on anxiety, depression, stress, and pain regulation^18,25,29–33^. However, the role of mindfulness meditation in cooperation has not been well investigated. Therefore, in the present study we explored the effect of mindfulness meditation on charitable giving as a real-world manifestation of human altruism relevant to large scale social cooperation.

Study participants recruited online were assigned randomly to treatment or control. In the treatment condition, participants watched a short mindfulness meditation video and were given an option to donate any part of their participation payment to a charity, whereas in the control condition, participants watched a drawing instruction video instead. Given a charity organization has little chance to directly, financially benefit our study participants, this type of giving measures a general form of altruism and is similar to that of cooperation in Dictator Game.

## Results

Among the 326 study participants, there are no significant differences in gender, age, highest educational attainment, and race and ethnicity between the two experimental groups (Table 1). 100% of the study participants correctly identified the content of the video, 94.5% correctly identified the event in the video. Most participants (79.6%) reported they watched the video until the end, while further analysis of time stamps showed that only 8.3% spent less than 1 minute on the video page (they did not watch the video for the full length) and only 4% spent more than 10 minutes on the video page (they were not watching the video when it finished) which indicates that a majority of the participants were compliers for the study (mean = 318 seconds, SD = 225). We observed no differences between the treatment and control groups.

**Table 1:** Distribution of Participants.

Category	Meditation	Drawing	*P*-value
All, N(%)	164 (50.3%)	162 (49.7%)	
Gender, N(%)			0.93
Male	110 (33.7%)	105 (32.2%)	
Female	54 (16.7%)	56 (17.2%)	
Age, Mean (SD)	32.7 (8.8)	34.0 (9.5)	0.12
Race, N(%)			0.44
White/Caucasian	124 (38.0%)	113 (34.7%)	
Asian	21 (6.4%)	27 (8.3%)	
Black/African American	9 (2.8%)	12 (3.7%)	
Hispanic	20 (6.1%)	19 (5.8%)	
Other	10 (3.1%)	9 (2.8%)	
Highest Educational Attainment, N(%)			0.93
High school	27 (8.3%)	27 (8.3%)	
Some College (1–3 years)	47 (14.4%)	52 (16.0%)	
College (4 years)	70 (21.5%)	66 (20.2%)	
Masters	17 (5.2%)	13 (4.0%)	
Other	3 (0.9%)	3 (0.9%)	

**Figure 1:**
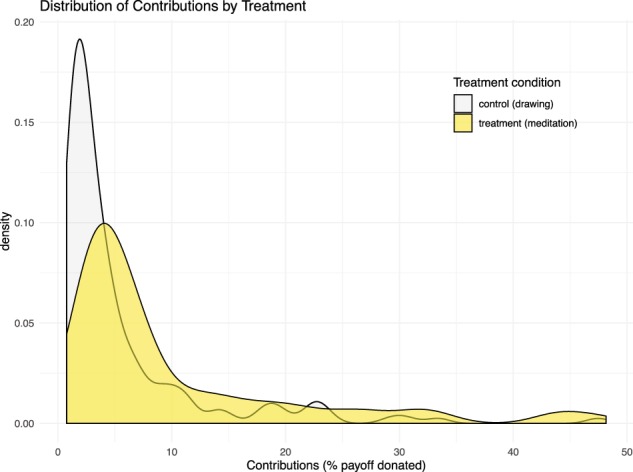
Distributions of contributions in the mindfulness meditation treatment group versus the control. Notes: Predicted contribution levels using the negative binomial regression model that controls for gender, age, education, race and ethnicity, and geography.

Level of donation different between the treatment and the control. The adjusted average donations of the subjects exposed to mindfulness mediation were 10.96% (SE = 1.20) of their endowment compared to 6.09% (SE = 0.81) of the endowment donated by the subjects in the control condition. Those who watched the mindfulness meditation video were found to give at a 2.61× greater rate than those in the control group, as a proportion of their participation payment, adjusted for covariates (p = 0.005). Regression results are reported in Table 2, and Fig. 1 displays predicted distribution of contributions in treatment and control based on the regression results. Among the control variables included, one year of age was associated with 7.47% higher rate of donations (p < 0.001), Hispanic subjects within the US donated at a 5.53 times greater rate than others (p < 0.001), everyone in the US donated an at average 48.94% lower rate than others (p = 0.04), and those in India donated at an average 4.94 greater rate than the rest (p < 0.001).

**Table 2:** Estimated treatment effect of meditation on contribution levels.

	Donation (% endowment)	
	IRR	(SE)
Treatment	2.61	(0.96)*
Age	1.07	(0.02)**
Female	1.67	(0.59)
Went to college	0.84	(0.36)
Black	1.84	(1.18)
Hispanic	5.53	(1.44)**
US	0.51	(0.17)*
India	4.94	(1.01)**
Constant	0.33	(0.18)
N	326	

Notes: *p < 0.05, **p < 0.01, ***p < 0.001. Negative binomial regression model. DV is % payoff donated by a subject. Reported incidence rate ratios and their standard errors adjusted for heteroskedasticity and geographic clusters. Black and Hispanic is indicated only if the subject is in the US, otherwise zero. US and India are as additional controls since they represent the two countries with the most participants.

**Figure 2:**
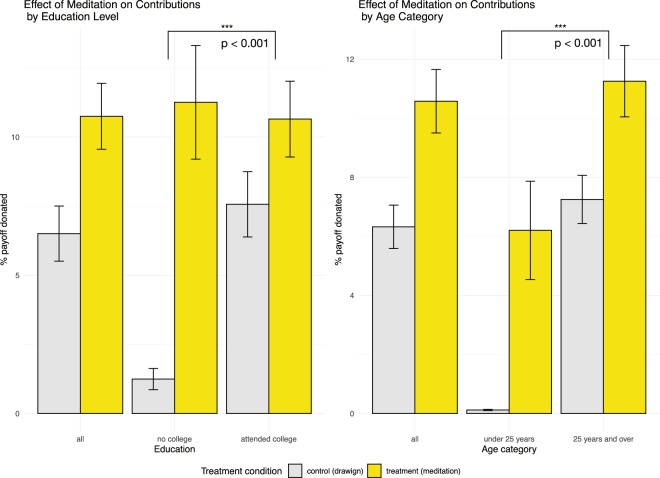
Interaction effects of education and age with the effect of mindfulness meditation treatment on contributions. Notes: (**A**) The difference in average contributions between the subjects exposed to mindfulness meditation and those in the control condition is significantly larger among subjects who did not attend college than in those who did. (**B**) The difference in average contributions between the subjects exposed to mindfulness meditation and those in the control condition is significantly larger in the younger group (under 25 years of age) than in the older group (25 years of age and older). In both cases, mean contribution levels and standard errors (adjusted for clustering by region) estimated using the negative binomial regression model with interaction effects (separately for the two cases) and controlling for gender, age, education, race and ethnicity, and geography.

Furthermore, we found significantly higher effects of mindfulness meditation on contributions in younger subjects (p < 0.001) and in those with lower educational attainment (p < 0.001) (Fig. 2). Examining the interaction effect of education using a regression model with an interaction term (which models education as a moderating factor), we found that in the group of subjects who never went to college, mindfulness meditation exposure translates to a jump from 1.25% (SE = 0.38) of the endowment donated in the control to 11.25% (SE = 2.05) of the endowment donated in the treatment condition. In contrast, among those who went to college, mindfulness mediation exposure translated to an increase from 7.57% (SE = 1.18) of the endowment donated in the control to 10.65% (SE = 1.37) of the endowment donated in the treatment condition. Examining the interaction effect of age group using the same regression framework that models moderation effect as an interaction term, we found similarly disproportionate patterns. In the under-25 age group, we found that mindfulness meditation exposure translated to a jump from 0.12% (SE = 0.02) of the endowment donated in the control to 6.20% (SE = 1.66) of the endowment in the treatment condition; and in 25-and-over age group the difference was between 7.57% (SE = 1.18) of the endowment donated in the control and 10.65% (SE = 1.37) of the endowment donated in the treatment condition. In estimating these averages, our models included all the controls described above; in particular, we controlled for age (in years) in estimating the treatment effects of both education group and age group, and we controlled for education in estimating the interaction effect of age group.

## Discussion

The present study found that individuals who participated in mindfulness meditation donated more than those who did not. The current findings are the first to identify a relation between mindfulness meditation and cooperation. The results imply potential great societal benefits. A mindfulness meditation session, even a short online one, can boost fundraising by non-profit organizations or for non-profit causes by companies. The study results also raise the question of whether human altruism is characterized not only by context modularity, where external factors (over which any one individual has little immediate control) influence its expression, but also whether humans have evolved capacity to upregulate or downregulate altruism, once taught to be a rigid individual or cultural characteristic, promoting cooperation when it is in the interest of collective survival.

Exposure to mindfulness meditation had an amplified treatment effect in a lower-educated group (subjects who never attended college) and in a younger group (subjects under 25 years of age). Our results confirm that older individuals on average are more likely to cooperate, and that the same is likely true of those with higher educational attainment (separate from the effects of mindfulness mediation). But we also offer evidence that younger subjects and those without any college education are more susceptible to our mindfulness mediation intervention designed to activate altruism and increase contribution levels. This in large part may be due to the fact that those same groups of subjects make very low contribution levels at the baseline to start with (i.e. in the control condition). While we estimated our models separately controlling for potential confounding of age and education, the differential treatment effects we observe in these two cases of interactions run parallel with each other, and may be capturing a single phenomenon that underlies both aging and education. Nevertheless, our results suggest that short-term mindfulness meditation has profound effects on subjects, managing to promote charitable giving across the board but especially in these relatively un-cooperative groups at the baseline level. The result raises the possibility that mindfulness meditation can upregulate human altruism and consequently modify individual decision-making, promoting cooperation.

A limitation of our study is the number of participants (N = 326). However, this sample size is multiple times larger than past studies examining the effect of meditation on social decision making (e.g. N = 49^34^, 59^35^, 39^36^, 101^37^, and 72^23^). In addition, our subjects are generally representative of the US population and beyond rather than of a socioeconomically homogenous group. The present study identifies most precisely the positive effect of mindfulness mediation in the general population. Exploring neurophysiological differences in these groups response to mindfulness mediation will advance our understanding of neurobiology of social cooperation, while broadening our subject population to increase diversity and not only sample size can help us design real-world approaches to promoting cooperation in an increasingly divided world.

## Methods

### Study participants

We recruited 354 Amazon Mechanical Turk (Mturk) workers from August, 2016 to November, 2018. These workers are individuals registered on the Mturk website that accept a variety of small jobs for monetary compensation, and represent the broad population of the US^38,39^. We eliminated 28 instances of duplicate participants from the study, leaving us with a final n = 326.

### Experimental design

Video Study participants were asked to watch one of two publicly available videos (our treatment was a mindfulness meditation practice video titled, “Breathing Meditation | UCLA Mindful Awareness Research Center”: https://www.youtube.com/watch?v=YFSc7Ck0Ao0 and our control was an instructional drawing video titled, “Drawing: How To Draw Mickey Mouse Step by Step! For kids!”: https://www.youtube.com/watch?v=EjMgBfNKjak). The mindfulness meditation video was created by Diana Winston, the director of Mindfulness Education at the UCLA Mindful Awareness Research Center with more than 20 years of experience as a mindfulness trainer and educator. The drawing video is not related to meditation and is not a video intended for relaxation either. Both videos are about the same length (4.5–5.5 minutes long). We randomly assigned our subjects to the meditation video (N = 164) and the other half to the control drawing instructional video (N = 162). We also had a time stamp program running in the background as a means of checking if each subject watched the entirety of the video properly.

#### Verification

After watching the assigned video, study participants were asked verification questions including “What was the video about? (Drawing instruction, Relaxing/meditation, Car, or Marathon)”; “Did you see somebody’s hand in the video? (Yes or No)” (a hand only appears in the drawing video); and “Did you do the activity that the video instructed? (Yes, until the end, Yes, but ceased in the middle, or No).” The questions asking about content serve the purpose of confirming that the study participants watched the online material. The question about doing the activity serves to check if participants did the activity specified in the video.

#### Social demographics

After answering the verification questions, participants were asked to answer demographic questions which asked them about their sex, age, race and ethnicity, and the highest educational attainment.

#### Donation

After providing the sociodemographic data, participants were informed of the amount of money they would receive ($1, $2, or $3). The amount was randomly chosen. Then, they were shown the message: “You can now make a charity donation to *United Way*. Please enter an amount between $0.00 and $X.00”, where $X.00 is the amount earned through participation in the survey. *United Way* (www.unitedway.org) is a non-profit organization (NPO) which helps people of low socioeconomic levels with health, education, and financial stability; it is a charity with which the authors have no conflict of interest.

### Data analysis

To analyze the effects of mindfulness meditation on contribution by our population, we estimated a negative binomial model, accounting for left-skewness of the distribution of contributions towards zero. Contribution in this context is the percent of their participation payoff that participants donated to *United Way* when prompted to do so. In our model we control for a gender, age, education, ethnicity, race and geography. Incidence rate ratios (IRRs) and standard errors adjusted for heteroskedasticity and clustering by regions are reported. In addition, we estimated two kinds of interaction effects: (1) between the meditation treatment and whether subjects attended college, and (2) between the meditation treatment and whether the subjects were younger than 25 years of age, or 25 and older (same controls included). This analysis also used the same regression model, but age or education were introduced separately as a moderating factor in the form of an interaction term with treatment. We took region into account because people’s behaviors and response to the mindfulness meditation practice are to a certain degree dependent on the specific culture of their surroundings. Although we expected some of the study participants would not watch the assigned video as we intended, we followed our statistical analysis plan (formulated before the experiment implementation to limit post-hoc explanation in favor of unbiased and clear hypothesis testing), performed intention-to-treat analysis and reported the results.

### Ethics Statement

The Yale University and the UCLA Human Subjects Committees approved this study, and waived the need for written informed consent from the participants. The methods were carried out in accordance with the relevant guidelines.

## Data Availability

The data reported in this paper are archived at Yale Institute for Network Science Data Archive and are available upon request.
